# Emotional Coherence Under Hypoxia and Aging: A Registered Report on Subjective–Physiological Coupling

**DOI:** 10.1111/psyp.70348

**Published:** 2026-07-12

**Authors:** Pierrick Laulan, Grégoire Millet, Ulrike Rimmele

**Affiliations:** ^1^ Institute of Psychology University of Lausanne Lausanne Switzerland; ^2^ Institute of Sport Sciences University of Lausanne Lausanne Switzerland

**Keywords:** aging, emotional coherence, heart‐rate variability, hypoxia, interoception, skin conductance

## Abstract

Emotional episodes involve partially coordinated changes in experience and autonomic activity, and subjective–physiological coherence appears to vary with aging and physiological stress. Physiological accounts of emotional aging propose that age‐related changes in autonomic and interoceptive function reduce the informativeness of bodily signals, weakening the coupling between feelings and bodily responses. We test whether subjective–physiological coherence is lower in older vs. younger normoxic adults, lower in hypoxic vs. normoxic younger adults, and similar in hypoxic younger adults and older normoxic adults; in secondary mechanism‐oriented analyses, we examine whether heart‐rate variability and cardiac interoceptive accuracy are statistically related to group differences in coherence. We will test these questions in three groups (*N* = 120): younger adults in normoxia, younger adults under acute hypoxia (FiO_2_ ≈ 12%), and older adults in normoxia. Participants will view emotional images and provide trial‐wise ratings of affect and approach motivation while heart rate and skin conductance are recorded. Emotional coherence will be indexed primarily by within‐person correlations between subjective arousal ratings and autonomic reactivity, with approach motivation and valence examined as complementary subjective dimensions.

## Abstract 2

This Stage 1 Registered Report tests a core but underexamined assumption in affective psychophysiology: that subjective bodily feelings covary with autonomic responses within individuals. Using acute hypoxia as an experimental perturbation and aging as a convergent comparison, the study provides a causal test of the acute hypoxia effect together with a convergent lifespan comparison of subjective–autonomic coherence. Pre‐registered trial‐level models and robust signal processing will clarify when coherence strengthens, weakens, or decouples, informing theories of emotion–body integration.

## 1 Introduction

Emotional episodes involve coordinated changes in subjective experience, physiology, and behavior (e.g., Scherer 2005). Emotional coherence refers to the covariation among these components within an emotional episode. Constructionist and interoceptive models propose that conscious feelings emerge when the brain integrates interoceptive signals with situational cues, goals, and conceptual knowledge (Barrett 2017; Lindquist 2013; MacCormack and Lindquist 2017). From this perspective, emotional coherence is expected to be context‐dependent and typically modest, because it depends on how bodily signals are attended to, interpreted, and weighted in affective inferences. Empirical work suggests that coherence is not uniform across response domains: different pairings (e.g., subjective–autonomic, subjective–expressive, or expressive–physiological coupling) can show different levels of coherence (Mauss et al. 2024). Coherence has also been examined across emotional versus non‐emotional states and under regulation manipulations (Constantinou et al. 2023; Dan‐Glauser and Gross 2013). Person‐level correlates such as body‐awareness training, emotion intensity, and habitual expressive suppression have also been examined in relation to coherence (Brown et al. 2020; Sze et al. 2010). By contrast, evidence that coherence differs systematically across specific discrete emotions remains limited and mixed (Mauss et al. 2005, 2024).

In this study, we focus on subjective–autonomic coherence: the within‐person covariation between self‐reported affect and autonomic reactivity across repeated emotional events. We treat this form of coherence primarily as a within‐person coupling process rather than as a trait‐like individual characteristic. Laboratory studies in young adults report small‐to‐moderate correlations between subjective affect and indices such as heart rate and skin conductance (Mauss et al. 2005; Sze et al. 2010). Although effect sizes are modest, individual differences in subjective–physiological coherence, indexed as stronger within‐person coupling within a given task or assessment context, have been associated with higher well‐being and more adaptive emotion regulation (Brown et al. 2020; Sommerfeldt et al. 2019), and coherence is sensitive to emotion‐regulation strategies, with suppression reducing and acceptance preserving coupling (Dan‐Glauser and Gross 2013). Characterizing when subjective–autonomic coherence is preserved or disrupted therefore has theoretical, applied, and potential clinical relevance.

Aging provides a natural context in which emotional coherence may change. Older adults often report higher positive affect and lower negative affect than younger adults despite accumulating health challenges, a pattern sometimes described as an aging paradox (Mather and Carstensen 2005; Mather 2024). Consistent with this view, recent image‐viewing work showed that older adults reported more positive and negative emotional sensations than younger adults, with a predominance of positive sensations, as well as more intense emotional sensations across image categories and greater emotional complexity particularly for negative images (Laulan and Rimmele 2024a). At the same time, aging is accompanied by alterations in cardiovascular and autonomic function, including reduced heart‐rate variability (HRV; Laulan and Rimmele 2026; Stein et al. 2008) and changes in baroreflex sensitivity (Maxwell et al. 2022). Vagal components of HRV are commonly interpreted as markers of autonomic capacity and flexibility (Laborde et al. 2017; Tuck et al. 2016). The Physiological Hypothesis of Emotional Aging (PHEA) proposes that age‐related changes in peripheral physiology and interoception make bodily signals noisier and less precise, reducing their impact on emotional inferences (MacCormack et al. 2023; Pfeifer and Cawkwell 2025). This view extends earlier maturational‐dualism proposals suggesting that age‐related changes in peripheral physiology and bodily perception may weaken the contribution of bodily activation to emotional experience (Mendes 2010). Together, these accounts imply that reduced coherence in older adults may reflect weaker trial‐level coupling between subjective experience and autonomic reactivity, but also that such coupling should be interpreted in relation to the overall profile of subjective and physiological reactivity. The empirical picture is heterogeneous: some interoceptive abilities decline with age while others remain stable or even improve, and the link between interoceptive sensitivity and affective outcomes shifts across the lifespan (Haustein et al. 2024; Khalsa et al. 2009; Mikkelsen et al. 2019; Murphy et al. 2018; Ulus and Aisenberg‐Shafran 2022).

Empirical evidence for age‐related changes in emotional coherence is beginning to accumulate but remains limited. Film‐based studies suggest that older adults can report equal or greater subjective arousal than younger adults while showing attenuated physiological responses, consistent with a partial decoupling of feelings from autonomic activation (Smith et al. 2005; Fernández‐Aguilar et al. 2020). However, few studies have used standardized, multi‐trial paradigms optimized for estimating individual differences in coherence, and existing findings vary across response systems and tasks (Brown et al. 2020; Sato and Saito 2024). Importantly, age effects on coherence may be emotion‐specific: Lohani et al. (2018) found that older adults showed greater coherence than younger adults during sadness, and older adults also showed greater physiological reactivity to sadness in loss‐related film paradigms (Seider et al. 2011). Such findings suggest that age‐related reductions in coherence should not be expected uniformly across all emotional contexts. One plausible boundary condition is arousal: according to the Strength and Vulnerability Integration (SAVI) model, age‐related physiological vulnerabilities should be most evident under high and sustained distress, whereas lower‐arousal or loss‐relevant emotions such as sadness may show different patterns (Charles 2010; Charles and Piazza 2024). PHEA therefore predicts reduced subjective–physiological coherence in older adults, but current empirical support is uneven and likely moderated by the emotional content of the elicitor.

Beyond aging, acute hypoxia offers a complementary, experimentally controllable way to perturb bodily states. Reducing inspired oxygen to FiO_2_ = 11%–13% (≈4000 m altitude equivalent) reliably lowers arterial oxygen saturation (SpO_2_) and can alter autonomic regulation in young adults (Bärtsch and Gibbs 2007; Taralov et al. 2015). Meta‐analytic evidence indicates that acute hypoxia in this range can impair reaction time, accuracy, and memory (McMorris et al. 2017; Ramírez‐delaCruz et al. 2024), and hypoxia has been associated with increased fatigue and less favorable affective responses, especially in prolonged or combined‐stressor paradigms (Stavrou et al. 2018). From an interoceptive standpoint, hypoxia simultaneously perturbs peripheral physiology and alters the cardiorespiratory afferent signals available to the brain, including carotid‐body chemosensory input about arterial oxygen availability (Burtscher et al. 2022; Kumar and Prabhakar 2012). If emotional coherence depends on the reliability and weight of these signals (Sze et al. 2010; Brown et al. 2020; Mauss et al. 2024), then acute hypoxia may transiently weaken the coupling between subjective affect and autonomic responses. Supporting this possibility, recent evidence from a hypoxia‐related clinical context indicates that oxygen saturation positively predicts heartbeat detection performance (Çaman et al. 2024). At the same time, associative memory for emotional material appears preserved under moderate normobaric hypoxia but becomes selectively disrupted at higher, hypobaric altitudes, with neutral scenes sometimes recognized more accurately than negative ones (Gatti et al. 2024), underscoring that hypoxia can reshape emotional–cognitive processing in nuanced ways.

Hypoxia and aging may converge on reduced emotional coherence through one candidate pathway: autonomic and interoceptive regulation. Aging is associated with progressive changes in autonomic flexibility, peripheral physiology, and body–brain signaling (MacCormack et al. 2023; Mather 2024; Maxwell et al. 2022), whereas acute normobaric hypoxia transiently perturbs oxygenation, autonomic control, and cardiorespiratory afferent signals (Bärtsch and Gibbs 2007; Burtscher et al. 2022; Taralov et al. 2015). This pathway is relevant to subjective–autonomic coherence because vagally mediated cardiac activity has been linked to cardiac interoceptive accuracy (Lischke et al. 2021) and implicated in emotion regulation (Laborde et al. 2017; Williams et al. 2015). Beyond this partial overlap, the two conditions diverge substantially: aging reflects decades of vascular, neural, motivational, and cognitive change (MacCormack et al. 2023; Mather 2024), whereas acute hypoxia is a short‐term, reversible perturbation of oxygen availability and cardiorespiratory regulation (Bärtsch and Gibbs 2007; Burtscher et al. 2022). Socioemotional selectivity theory (SST) raises a second, more speculative possibility: acute threats to bodily resources such as oxygen scarcity could narrow future time perspective and shift priorities toward emotionally meaningful information (Mather and Carstensen 2005; Scheibe and Carstensen 2010). To our knowledge, the only experimental test of this idea in a hypoxic context, using small samples under normobaric and hypobaric hypoxia, did not find the predicted preferential retention of positive over negative material (Gatti et al. 2024), so any motivational contribution remains uncertain. We therefore treat convergence between older adults and hypoxic younger adults as evidence consistent with partial overlap on a bodily‐signal pathway, not as evidence that acute hypoxia reproduces the broader vascular, neural, motivational, or cognitive profile of aging.

In the present study, we adopt a three‐group design to test whether aging and acute hypoxia reduce subjective–autonomic coherence, thereby testing one prediction derived from PHEA. Younger adults under normoxia, younger adults under acute normobaric hypoxia (FiO_2_ ≈ 12%), and older adults under normoxia will view emotional pictures and rate each image on valence, arousal, and approach motivation. Stimuli span a broad affective range, with emotional images normatively more arousing than neutral images, so that coherence can be assessed across varying levels of emotional engagement. We operationalize subjective–autonomic coherence as the within‐person covariation across trials between subjective arousal and autonomic responding (heart‐rate change and skin conductance), adapting prior within‐person approaches that model trial‐level/stimulus‐level associations or within‐subject covariation across emotional stimuli (Cuve et al. 2023; Rattel et al. 2020). Event‐related heart‐rate change and skin conductance responses are widely used autonomic indices of emotional engagement during picture viewing (Lang et al. 1993; Bradley et al. 2001; Kreibig 2010). We will collect valence, arousal, and approach motivation to capture complementary facets of subjective experience, but the primary coherence analyses operationalize subjective affective intensity as arousal/activation, because arousal is the most established subjective dimension for indexing affective intensity in picture‐viewing paradigms and is particularly closely linked to electrodermal response magnitude. Approach motivation and valence are retained as complementary dimensions and examined in exploratory component‐level analyses. To contextualize coherence effects, we will also report mean subjective arousal and mean autonomic reactivity by group. This will allow us to determine whether any reduction in coherence is accompanied by attenuated raw subjective or physiological reactivity, or whether coherence differences emerge despite preserved or heightened mean arousal and autonomic responses. Because, in the present protocol, older adults are not exposed to hypoxia for ethical and risk‐management reasons, the design supports two direct contrasts (i.e., older vs. younger adults under normoxia, and hypoxic vs. normoxic younger adults) but cannot estimate additive or interactive Age × Oxygen effects. This choice reflects the cardiovascular and respiratory demands of acute hypoxic exposure and the greater health‐risk heterogeneity of older samples; we acknowledge, however, that age alone is not a universal contraindication to hypoxia in healthy, well‐screened individuals (e.g., Parati et al. 2018; Raberin et al. 2023). Any comparison between hypoxic younger adults and normoxic older adults is therefore interpreted as an outcome‐level partial‐convergence comparison.

Our primary hypotheses are that (H1, Aging) older adults will show lower average subjective–physiological coherence than younger adults in normoxia, consistent with PHEA; (H2, Hypoxia effect) acute hypoxia will reduce average coherence in younger adults relative to normoxia; and (H3, partial convergence) coherence in hypoxic younger adults will be numerically similar to that of older adults, consistent with an autonomic–interoceptive overlap. In secondary, mechanism‐oriented analyses, we will examine whether group differences in coherence are statistically related to resting vagal activity and objective cardiac interoceptive accuracy. Because acute hypoxia can impair cognitive performance, particularly response speed and accuracy, and may also affect attention, fatigue, and affective state depending on exposure severity, duration, and task demands (McMorris et al. 2017; Ramírez‐delaCruz et al. 2024; Stavrou et al. 2018), these factors could in principle influence the quality and consistency of subjective ratings. Such effects could therefore contribute to group differences in subjective–physiological coherence independently of, or in addition to, the proposed bodily‐signal pathway. We therefore include state and response‐quality measures to aid interpretation of any observed group differences. Beyond the primary, valence‐collapsed hypotheses, we will exploratorily examine whether age‐ and hypoxia‐related differences in coherence vary by stimulus valence. Previous work suggests that age effects on coherence and reactivity can be emotion‐specific, with relatively preserved or even heightened experience–physiology coherence for sadness in older adults when loss‐related films are used (Lohani et al. 2018; Seider et al. 2011) and more heterogeneous patterns for other emotions. Given these mixed findings, we do not formulate strong directional predictions by valence and treat these analyses as exploratory.

## 2 Methods

### 2.1 Open Science and Stage 1 Status

This article is a Stage 1 Registered Report. The full protocol, analysis scripts, and anonymized data will be made available on the Open Science Framework (OSF) upon completion of data collection and initial analyses. All hypotheses, inclusion/exclusion criteria, preprocessing steps, and statistical models described below are preregistered and will be implemented as specified. Any deviations will be transparently documented and labeled as exploratory.

### 2.2 Participants

We will recruit 120 community‐dwelling adults: 80 younger adults (18–35 years) and 40 older adults (60–80 years). Younger adults will be randomly assigned to a normoxia group (*n* = 40) or a hypoxia group (*n* = 40). Older adults will be tested only under normoxia (*n* = 40). Within each group, we will aim for balanced gender representation. Participants will be recruited via university mailing lists, local advertisements, and community organizations. Eligible participants will be community‐dwelling adults aged 18–35 years (younger group) or 60–80 years (older group), fluent in French, with normal or corrected‐to‐normal vision and right‐handed. All participants must provide written informed consent and must not have stayed more than 48 h above 2000 m in the preceding 6 months, in order to limit interindividual variability related to recent altitude acclimatization, which can persist after return to lower altitude and modulate physiological responses to hypoxia (e.g., Burtscher et al. 2022; Treml et al. 2020).

For older adults, global cognition will be screened with the French version of the Telephone Interview for Cognitive Status (F‐TICS‐m/TICS‐F; Lacoste and Trivalle 2009; Vercambre et al. 2010). The TICS‐m family of instruments is a brief, validated measure of global cognitive functioning designed for epidemiological screening (Knopman et al. 2010). We will include only older participants with a score > 27 (on the 0–41 scale used here), using ≤ 27 as a conservative screening‐based exclusion threshold intended to reduce the likelihood of including participants with major cognitive impairment. This threshold is an eligibility criterion and will not be interpreted as a clinical diagnosis (Knopman et al. 2010).

Exclusion criteria focus on minimizing risk under acute normobaric hypoxia and ensuring interpretable autonomic recordings. Potential participants will be excluded if they report past or current cardiovascular, respiratory, neurological, metabolic, or major psychiatric disorders; uncontrolled hypertension; a history of altitude‐related illness requiring medical attention; diagnosed sleep apnea, chronic obstructive pulmonary disease, or other chronic pulmonary disease incompatible with hypoxic exposure; conditions incompatible with repeated non‐invasive blood pressure monitoring; current use of medications known to substantially affect cardiovascular, respiratory, autonomic, or neuroendocrine function (e.g., β‐blockers, non‐stabilized antihypertensives, systemic corticosteroids, psychostimulants); current smoking or vaping; or body‐mass index (BMI) ≥ 32. These criteria are consistent with safety recommendations for short‐term exposure to normobaric hypoxia in research and therapeutic contexts, which emphasize careful screening for cardiopulmonary and vascular disease and close monitoring of at‐risk individuals (e.g., Burtscher et al. 2012; Millet et al. 2016). Pregnancy or suspected pregnancy is a contraindication to hypoxic exposure; women of childbearing potential will therefore be screened for pregnancy status at inclusion and re‐checked on the test day, and any reported or suspected pregnancy will lead to exclusion from the study.

### 2.3 Sample Size Justification

The sample size of 120 participants (40 per group) was determined primarily by resource constraints, consistent with a feasibility‐based approach to sample size justification (Lakens 2022). Recruitment of older adults for multi‐hour physiological testing combined with hypoxia safety protocols is logistically demanding, and the hypoxia facility has limited availability within the project timeline. *N* = 120 (40 per group) represents the maximum feasible sample within the hypoxic chamber, safety monitoring, recruitment, and 12‐month funding constraints of the project, and substantially larger samples are not achievable under these conditions. Following recommendations for resource‐constrained designs, we complement this feasibility‐based justification with a sensitivity analysis to clarify which effect sizes the study can detect with reasonable power, allowing readers and reviewers to judge the informativeness of the design given its constraints (Lakens 2022).

### 2.4 Sensitivity Analyses

Because closed‐form power calculations for generalized estimating equations (GEE) with non‐zero within‐subject correlation are not generally available in simple form and depend on design‐specific assumptions about the correlation structure (e.g., Gastañaga et al. 2006; Li and McKeague 2013), we based our sensitivity analyses on a two‐sample *t*‐test applied to person‐level coherence indices. This follows a pragmatic approach in which power is approximated for a simpler model that uses the same outcome and contrasts as the planned analysis (Frison and Pocock 1992). The resulting thresholds provide a transparent estimate of the smallest standardized between‐group differences that the design can detect. In the actual analyses, GEE models will use all available trials and account for within‐person dependence; modeling the correlation structure generally improves efficiency relative to analyses that ignore correlation (e.g., Li and McKeague 2013), so the *t*‐test–based sensitivity estimates can be regarded as conservative.

For H1 (older vs. younger adults in normoxia) and H2 (younger adults in hypoxia vs. normoxia), we treated the group difference in coherence as an independent‐samples *t*‐test on Cohen's d and used the *pwr* package (Champely 2020) in R with *α* = 0.05, *n* = 40 per group, and 80% power. Under these assumptions, the minimal detectable standardized difference is *d* = 0.63 for a two‐sided test. This effect size falls in the medium‐to‐large range (Cohen 1988) and indicates that the design is primarily sensitive to medium‐to‐large group differences in coherence. Although group‐level inference is constrained by the number of independent participants, the repeated trial‐level structure of the task provides substantially more information for estimating within‐person subjective–physiological coupling within each participant; this improves the precision with which individual coherence estimates can be derived but does not increase the number of independent units for between‐group inference. Smaller effects will be interpreted cautiously and may motivate a larger‐scale or multi‐site follow‐up if the observed pattern is theoretically informative but statistically unresolved.

For H3 (partial convergence), we are concerned with whether coherence in hypoxic younger adults is sufficiently similar to that of older adults to support an interpretation of partial overlap on the autonomic–interoceptive pathway. Given the planned sample size of *n* = 40 per group, standard equivalence tests on Cohen's d with theoretically meaningful bounds in the small‐to‐moderate range remain underpowered (Lakens 2017; Lakens et al. 2018). We therefore do not treat H3 as a fine‐grained equivalence test. For the equivalence procedure described in the Statistical analysis plan, we pre‐specify two bounds: a pragmatic design‐anchored sensitivity bound of *d* = 0.63, corresponding to the magnitude the present design is reasonably positioned to adjudicate, and a more conservative secondary bound of *d* = 0.35, informed by prior work on age differences in emotional coherence (e.g., Lohani et al. 2018) and by the small‐telescopes logic (Simonsohn 2015).

### 2.5 Hypoxia Apparatus and Safety Monitoring

Testing will take place in a normobaric hypoxic chamber at Centre Sport et Santé (CSS), Service Sport Santé UNIL+EPFL, Centre sportif de Dorigny, Lausanne, Switzerland. Inspired oxygen fraction (FiO_2_) will be controlled by a gas‐mixing hypoxic system (AltiTrainer, SMTEC SA, Nyon, Switzerland), which generates normobaric hypoxia by mixing nitrogen into ambient air and continuously regulates FiO_2_ via an inline oxygen sensor and PO_2_ probe. This system allows stable delivery of room air (FiO_2_ = 21%) for normoxic conditions and hypoxic air with a target FiO_2_ between 11.5% and 12.0% for the hypoxia condition (see Lichtblau et al. 2025).

SpO_2_ and heart rate will be monitored continuously via finger pulse oximetry throughout the session. Non‐invasive blood pressure will be measured at baseline and at regular intervals (every 10 min during acclimatization, then at midpoint and end of the task phase) using an automated cuff. Acute mountain sickness (AMS) symptoms (headache, nausea, dizziness) will be assessed repeatedly using a brief standardized checklist derived from the Lake Louise Score. These monitoring procedures and thresholds are consistent with current recommendations for short‐term normobaric hypoxia in healthy participants (e.g., Burtscher et al. 2022).

For younger adults assigned to the hypoxia condition, FiO_2_ will be gradually reduced during the acclimatization phase until SpO_2_ stabilizes between 75% and 85%, corresponding to a simulated altitude of approximately 3500–4000 m that is generally well tolerated under close monitoring. In the overall experimental session, this hypoxic (or normoxic) condition will first be established for a separate memory task from a companion project; the emotional image‐viewing task and the interoceptive accuracy task of the present study will then be administered within the same oxygen condition immediately afterward, without altering FiO_2_. Younger adults assigned to the normoxia condition and older adults tested under normoxia will remain at FiO_2_ = 21% throughout, with identical physiological and symptom monitoring but no hypoxic exposure.

The session will be immediately interrupted and the chamber returned to normoxia (FiO_2_ = 21%) if any of the following occur: SpO_2_ < 75% for more than 30 consecutive seconds; systolic blood pressure > 180 mmHg or diastolic blood pressure > 105 mmHg on two consecutive readings; sustained heart rate > 140 bpm for more than 2 min; or the participant reports severe headache, nausea, dizziness, chest pain, or marked distress. The session will also be stopped immediately if the participant requests to discontinue or if the experimenter or study physician judges continuation to be unsafe. The study physician will be available remotely on call during experimental sessions to provide medical advice or support if needed, including if symptoms do not resolve promptly after return to normoxia. For younger participants assigned to the hypoxia condition, total hypoxic exposure duration (from SpO_2_ stabilization to the end of the task phase) is expected to be approximately 70–80 min. As a manipulation check, we will verify that mean SpO_2_ during the emotional task is below 85% for hypoxia participants; individuals who do not reach or maintain this threshold will be flagged and reported.

### 2.6 Stimuli

Participants will view 120 images (40 negative, 40 neutral, 40 positive) taken from the stimulus set developed by Laulan and Rimmele (2024b). All images originate from the International Affective Picture System (IAPS; Lang et al. 2005). Normative valence and arousal ratings (1–9 scales) were taken from Leclerc and Kensinger (2011), who collected ratings from 50 younger and 50 older adults.

Based on the normative ratings, negative images have mean valence scores between 1 and 3.5 in both age groups, neutral images between 4 and 6, and positive images between 6.5 and 9. One‐way ANOVAs confirmed the expected effect of valence in younger adults, *F*(2, 117) = 1076.49, *p* < 0.001, and in older adults, *F*(2, 117) = 930.64, *p* < 0.001, indicating that negative, neutral, and positive images are clearly differentiated by valence in both groups. Arousal ratings show the typical U‐shaped pattern: in younger adults, negative and positive images are more arousing than neutral images, *F*(2, 117) = 83.85, *p* < 0.001, and the same pattern holds in older adults, *F*(2, 117) = 55.18, *p* < 0.001. Within each valence category, paired *t*‐tests on the per‐image differences between younger and older raters revealed no reliable age differences in valence or arousal (all |*t*(39)| ≤ 1.56, *ps* ≥ 0.12), ensuring that any age‐ or condition‐related effects in our task are not trivially explained by baseline differences in normative ratings.

To reduce potential confounding from low‐level visual properties, we ensured that negative, neutral, and positive images were matched on basic physical features using the quantitative image descriptors provided by Laulan and Rimmele (2024b). In the present 120‐image subset, luminance, contrast, entropy, and the L*, a*, and b* dimensions of CIELAB color space do not differ reliably across valence categories, all *Fs*(2, 117) ≤ 1.65, *ps* ≥ 0.20 (see Table 1 for descriptive statistics). We also verified that images are matched on semantic content: each image was coded as depicting people, faces, animals, objects, or landscapes, and a chi‐square test indicated that the distribution of content types does not differ across valence categories, *χ* ^2^(8) = 3.62, *p* = 0.89.

**TABLE 1:** Normative ratings and physical properties of the images by valence category.

Measure	Negative	Neutral	Positive	*F*(2, 117)	*p*
Valence (younger)	2.59 (0.47)	5.03 (0.36)	7.35 (0.53)	1076.49	< 0.001
Valence (older)	2.56 (0.50)	5.07 (0.47)	7.23 (0.48)	930.64	< 0.001
Arousal (younger)	5.20 (0.67)	3.38 (0.52)	5.03 (0.85)	83.85	< 0.001
Arousal (older)	5.07 (0.96)	3.27 (0.69)	4.95 (0.90)	55.18	< 0.001
Luminance	91.55 (33.95)	87.26 (35.09)	96.48 (39.87)	0.64	0.528
Contrast	70.10 (14.91)	72.12 (14.99)	67.67 (15.37)	0.87	0.420
Entropy	6.73 (1.10)	6.44 (1.18)	6.69 (1.23)	0.74	0.482
L*	37.97 (13.86)	36.34 (14.46)	40.43 (16.02)	0.77	0.463
a*	5.21 (6.60)	4.95 (7.05)	5.44 (8.87)	0.04	0.960
b*	9.77 (8.59)	14.30 (11.92)	12.59 (12.82)	1.65	0.196

*Note:* *N* = 120 images (40 negative, 40 neutral, 40 positive). Values are means with standard deviations in parentheses. *F* and *p* values correspond to one‐way ANOVAs testing the effect of valence category on each measure.

### 2.7 Emotional Image Task

#### 2.7.1 Trial Structure

The emotional image task consists of 120 trials (40 negative, 40 neutral, 40 positive). Each trial follows a fixed sequence designed to elicit reliable autonomic responses while allowing for precise trial‐by‐trial ratings, in line with standard affective picture‐viewing paradigms combining physiology and self‐report (e.g., Lang et al. 1993; Bradley et al. 2001). Each trial begins with a jittered pre‐stimulus fixation period of 5000–7000 ms, signaled by a central fixation cross on a neutral gray background. This fixation period reduces temporal predictability, allows partial recovery from responses elicited by the preceding trial and rating phase, and provides an analytically usable local pre‐stimulus electrodermal baseline; the final 2 s of the fixation period will be used to derive local pre‐stimulus skin conductance level (SCL) for tonic‐adjusted skin conductance response (SCR) analyses (see electrodermal‐activity preprocessing). The fixation is followed by an image presentation period of 6000 ms, placing the primary SCR scoring window entirely within the image‐viewing period and before the rating phase. Heart rate and skin conductance are recorded continuously throughout the fixation and image epochs.

Immediately after image offset, participants complete three self‐report ratings presented sequentially on the screen: valence, arousal, and approach motivation. Each visual analog scale (VAS) is introduced by an explicit prompt: “While viewing the image you just saw, how negative or positive did you feel?”; “While viewing the image you just saw, how activated or aroused did you feel?”; and “While viewing the image you just saw, to what extent did you feel a tendency to avoid or approach what was shown?”

Each rating is given on a VAS ranging from 0 to 100, displayed as a horizontal line with verbal anchors at the endpoints (and midpoint when appropriate). For valence, the scale is anchored at “very negative” (0), “neutral” (50), and “very positive” (100), similar to prior work using VAS‐based affect ratings in image‐viewing and reappraisal tasks (e.g., Pollatos et al. 2007). Arousal is rated from “not at all activated” (0) to “very activated” (100). Approach motivation is rated from “strong tendency to avoid” (0), through “no tendency either way” (50), to “strong tendency to approach” (100), consistent with conceptualizations of approach–avoidance motivation as graded tendencies to move toward vs. away from stimuli (Elliot 2006; Harmon‐Jones et al. 2013). Participants move a cursor along the VAS using response keys and confirm their choice with a button press; each scale remains on screen until response, up to a maximum of 9000 ms. If no response is made within this window, the trial advances and the missing value is recorded. Participants will respond to the rating prompts using the hand opposite to the electrodermal activity (EDA) recording hand and will be explicitly instructed to keep the EDA hand still throughout the task to minimize movement artifacts. Onset and offset of fixation, image presentation, each rating prompt, and each rating response will be marked with synchronized triggers in the physiological recording stream to support precise window definition during preprocessing.

Rating latencies and omission rates will be retained as task‐embedded indicators of response quality and attentional engagement. This approach is motivated by evidence that acute hypoxia can slow response speed and impair cognitive performance (McMorris et al. 2017; Ramírez‐delaCruz et al. 2024), and by vigilance research showing that reaction‐time slowing and omission‐like lapses are sensitive markers of reduced alertness in dedicated psychomotor vigilance tasks (Basner et al. 2011; Dinges and Powell 1985). These indices will be analyzed in planned sensitivity analyses to determine whether group differences in subjective–physiological coherence are robust to variation in response quality and attentional engagement. Stimuli are presented in pseudo‐random order with the constraint that no more than three images of the same valence appear consecutively. The task is divided into three blocks of 40 trials each, with brief self‐paced breaks between blocks. Total task duration is approximately 35–40 min.

#### 2.7.2 Subjective Indices

For each trial, we will extract three subjective indices: valence, arousal, and approach motivation. For the primary coherence analyses, subjective affective intensity will be operationalized as the arousal rating because perceived activation is the subjective dimension most directly aligned with affective intensity in picture‐viewing paradigms and is particularly closely linked to electrodermal response magnitude (Lang et al. 1993; Bradley et al. 2001; Kreibig 2010). Arousal ratings will be standardized within person across valid emotional trials before coherence estimation.

Approach motivation and valence will be retained as complementary subjective dimensions. To examine the strength of action tendency independently of its direction, we will compute motivational intensity as the absolute deviation of approach‐motivation ratings from the neutral midpoint, |approach −50|. Valence ratings will be retained as a separate subjective dimension because they index affective direction, which need not covary monotonically with autonomic response magnitude. As exploratory component‐level analyses, we will also compute motivational‐intensity coherence and valence‐intensity coherence, with valence intensity defined as the absolute deviation from neutral valence, |valence −50|. These component‐level analyses will be interpreted as exploratory tests of whether different subjective dimensions show distinct patterns of subjective–physiological coupling.

#### 2.7.3 Trait Emotion Regulation

Habitual emotion regulation will be assessed using the 10‐item Emotion Regulation Questionnaire (ERQ; Gross and John 2003; French version: Christophe et al. 2009), administered before chamber entry. The ERQ assesses two habitual emotion‐regulation strategies: cognitive reappraisal (6 items) and expressive suppression (4 items). Items are rated on a 7‐point Likert scale and averaged within each subscale. Internal consistency for both subscales will be reported in the present sample. The Suppression subscale is most directly relevant to subjective–physiological coherence, given prior evidence that suppression and related emotion‐regulation strategies can alter coherence between subjective experience and physiological responses (Brown et al. 2020; Dan‐Glauser and Gross 2013), but both ERQ subscales will be reported by group and used as sensitivity covariates if meaningful between‐group differences are observed (see Sensitivity analyses).

### 2.8 Trait Interoceptive Sensibility

In addition to trial‐wise ratings, participants will complete the Multidimensional Assessment of Interoceptive Awareness, Version 2 (MAIA‐2; Mehling et al. 2018; French validation: Da Costa Silva et al. 2022) before chamber entry. MAIA‐2 assesses several dimensions of self‐reported interoceptive sensibility, including noticing bodily sensations, sustaining attention to the body, linking bodily sensations to emotional experience, listening to the body for self‐regulation, and trusting bodily signals. We will compute the eight MAIA‐2 subscale scores according to the published scoring instructions and derive a prespecified global composite as the sum of the eight subscale means (range 0–40); we will also report the Emotional Awareness subscale because it is the facet most directly related to emotional awareness. In the main confirmatory analyses, MAIA‐2 will not be treated as a central predictor of coherence. Instead, the prespecified MAIA‐2 global composite and the Emotional Awareness subscale will each be included, when relevant, in separate sensitivity models to evaluate whether group differences in coherence are robust to stable interoceptive sensibility and bodily emotional awareness. Analyses of other individual MAIA‐2 subscales will be clearly labeled as exploratory.

### 2.9 Interoceptive Accuracy Task

To obtain an objective state measure of cardiac interoception under each oxygen condition, participants will complete a heartbeat discrimination task adapted from Garfinkel et al. (2015). During each trial, a sequence of 10 auditory tones (50 ms; 800 Hz) will be presented over headphones while electrocardiogram (ECG) is recorded and R‐peaks are detected online for tone triggering. On half the trials, the acoustic onset of each tone will occur 250 ms after the corresponding ECG R‐peak (conventionally synchronous condition). On the other half, the acoustic onset of each tone will occur 550 ms after the corresponding ECG R‐peak (conventionally asynchronous condition). After each tone sequence, participants will indicate whether the tones were “in sync” or “out of sync” with their heartbeat. Before the task, audibility of the 800‐Hz tones will be checked for each participant, and headphone level will be adjusted to a clearly audible and comfortable level and then kept constant throughout the task. Participants who cannot hear the tones clearly at a comfortable level will not complete the heartbeat discrimination task; their interoceptive‐accuracy outcome will be treated as missing, but they will remain eligible for the primary emotional‐coherence analyses.

The task will comprise 80 trials (40 synchronous, 40 asynchronous) presented in randomized order, with brief breaks after every 20 trials. Each trial will begin with a fixation cross (1000 ms), followed by the tone sequence (approximately 8–10 s, depending on heart rate) and a response screen (up to 4000 ms). A jittered inter‐trial interval (1000–2000 ms) will separate trials.

Heartbeat discrimination accuracy will be quantified using signal detection theory indices, with *d*ʹ computed separately for each participant based on hits (correct “in sync” responses on synchronous trials) and false alarms (incorrect “in sync” responses on asynchronous trials). Trials with missing responses or technical artifacts preventing verification of the prespecified R‐peak‐to‐acoustic‐onset delay (e.g., ECG dropout, erroneous online R‐peak detection, trigger loss, or audio‐output failure) will be excluded. A loglinear correction will be applied to all participants before calculating d′: H = (hits + 0.5)/(valid synchronous trials + 1), FA = (false alarms + 0.5)/(valid asynchronous trials + 1), and d′ = z(H) − z(FA), where z denotes the inverse cumulative standard‐normal function (Hautus 1995). The numbers of valid synchronous and asynchronous trials will be reported by group. This dʹ index will serve as our primary measure of interoceptive accuracy under each group's oxygen condition and will be used in mechanism‐oriented analyses of emotional coherence. As emphasized in contemporary interoception frameworks, heartbeat discrimination performance reflects not only the fidelity of cardiac afferent processing but also attentional, decisional, and metacognitive factors (Garfinkel et al. 2015; Murphy et al. 2018). We therefore interpret dʹ as discrimination performance between these prespecified delays and as an operational state marker of cardiac interoceptive accuracy in this task context, rather than as a pure measure of interoceptive ability.

### 2.10 Physiological Recording and Preprocessing

#### 2.10.1 Cardiac Measures (ECG, Event‐Related Heart Rate, Resting HRV)

ECG will be recorded continuously throughout the session using a BIOPAC MP160 system (BIOPAC Systems Inc., Goleta, CA) with a three‐lead modified Lead II configuration and a sampling rate ≥ 1000 Hz. For the heartbeat discrimination task, R‐peaks will additionally be detected online from the ECG signal and used to trigger tone presentation. Before data collection, the complete R‐peak‐to‐acoustic‐onset pathway will be validated by recording the raw ECG, the online R‐peak trigger, and an audio‐loopback or microphone signal capturing the actual headphone output on synchronized channels; programmed delays will be adjusted so that measured acoustic onsets occur 250 and 550 ms after the raw ECG R‐peak. Signals will be acquired with AcqKnowledge software, band‐pass filtered using standard ECG settings, and stored for offline analysis. For offline analyses, R‐peaks will be re‐detected from the raw ECG using automated algorithms and visually inspected by trained staff; artifacts and ectopic beats will be corrected manually or, when necessary, excluded from analysis. Inter‐beat intervals (IBIs) will be exported for heart‐rate (HR) and HRV analyses.

#### 2.10.2 Event‐Related Heart‐Rate Change

For each trial of the emotional image task, we will compute event‐related HR change (ΔHR) as the mean HR during the 0–4 s interval after image onset (i.e., most of the image presentation period) minus the mean HR in a 1‐s pre‐stimulus baseline (−1 to 0 s). This window is chosen to capture the short‐latency orienting‐related deceleration and early modulation of HR during picture viewing while avoiding contamination by the subsequent rating phase, consistent with prior psychophysiological work on cardiac responses to affective pictures (e.g., Lang et al. 1993; Bradley et al. 2001). Trials with excessive artifacts in the analysis window (e.g., > 20% interpolated IBIs) will be discarded. For HR‐based coherence analyses, we will define a cardiac deceleration score as HRdec = −ΔHR = mean HR during the pre‐stimulus baseline minus mean HR during the 0–4 s post‐stimulus interval. Larger HRdec values therefore indicate greater stimulus‐related cardiac deceleration, whereas negative values indicate acceleration. HRdec will be used in all HR‐based coherence correlations and trial‐level GEE models; alternative response windows (e.g., 1–4 s) will be examined in sensitivity analyses.

#### 2.10.3 Resting Heart‐Rate Variability

Resting HRV will be assessed in two standardized 5‐min periods: (a) a seated normoxic baseline before entering the hypoxia chamber and (b) a seated resting period under each participant's assigned oxygen condition (normoxia vs. hypoxia) immediately before the task sequence conducted under that oxygen condition. During these recordings, participants will sit quietly, breathe spontaneously, and keep their eyes open. HRV will be derived from artifact‐corrected IBIs following current recommendations for experimental and clinical HRV assessment (Task Force of the European Society of Cardiology and the North American Society of Pacing and Electrophysiology 1996; Laborde et al. 2017). The primary index will be the natural‐log transformed root mean square of successive differences (lnRMSSD), a widely used proxy of vagally mediated cardiac control in psychophysiological research, while acknowledging ongoing debates about the specificity and reliability of HRV metrics (Laborde et al. 2017; Nicolini et al. 2024; Pinna et al. 2007). For mechanism‐oriented analyses, we will focus on lnRMSSD recorded under the assigned oxygen condition as a state‐dependent estimate of autonomic capacity in the context in which emotional coherence is assessed. Baseline normoxic lnRMSSD will be reported descriptively and used in sensitivity analyses to test whether group differences in coherence are better explained by state‐ versus trait‐like autonomic factors.

#### 2.10.4 Electrodermal Activity (Skin Conductance Responses)

EDA will be recorded using a BIOPAC EDA amplifier module connected to the MP160 system, with data sampled at ≥ 1000 Hz and downsampled offline as needed. Two Ag/AgCl electrodes filled with isotonic paste will be attached to the thenar and hypothenar eminences of the non‐dominant hand, following standard psychophysiological guidelines (Dawson et al. 2007). EDA signals will be low‐pass filtered at 5 Hz, visually inspected for artifacts, and segmented into trial‐wise epochs time‐locked to image onset.

Phasic SCR magnitude will be used as the primary electrodermal index of stimulus‐locked reactivity. For each trial, local pre‐stimulus SCL will be computed as the mean skin conductance level, after artifact rejection, during the final 2 s of the pre‐stimulus fixation period. Phasic SCR will be computed as the peak‐to‐baseline increase following image onset, using the final 1 s of the pre‐stimulus fixation as the local SCR baseline. The primary SCR scoring window will be 1–5 s after image onset, so that the SCR window remains entirely within the image‐viewing period and precedes the rating phase. This window lies within the commonly recommended range for stimulus‐evoked SCRs (onset latencies = 1–4 s, peaks = 3–6 s; Dawson et al. 2007; Boucsein et al. 2012; see also Benedek and Kaernbach 2010).

Following common electrodermal scoring practice (Dawson et al. 2007; Boucsein et al. 2012), SCR non‐responses, defined in the primary analysis as peak amplitude < 0.02 μS, will be coded as zero rather than treated as missing, so that SCR magnitude reflects both response occurrence and response size. Zero‐coded SCRs will be interpreted as the absence of a detectable stimulus‐locked phasic increase above the local baseline, not as evidence for the absence of electrodermal sympathetic engagement. This distinction matters because physiological non‐response is meaningful information about emotional engagement, whereas recording artifacts reflect data loss. To distinguish response occurrence from response size, SCR amplitude conditional on response detection will be summarized separately alongside SCR response frequency.

Because hypoxia may increase tonic electrodermal activation, tonic SCL will also be derived and summarized by group and block, alongside local pre‐stimulus SCL trial‐level distributions. These tonic measures are not part of the primary coherence outcome, which concerns stimulus‐locked phasic coupling. They will be used descriptively and in sensitivity analyses to assess whether elevated tonic sympathetic/sudomotor activation under hypoxia could reduce the detectability or dynamic range of phasic SCR responding (see Sensitivity analyses).

Trials affected by recording artifacts, excessive movement, signal loss, or unusable signal quality will be treated as invalid and excluded from analyses for the affected physiological modality. Chamber temperature, and when available relative humidity, will be recorded throughout each session and reported, because both can influence electrodermal activity (Boucsein et al. 2012). Trial‐wise SCR amplitudes will be used as the primary electrodermal component of subjective–physiological coherence.

### 2.11 Procedure

Eligibility screening will take place before the laboratory session, via telephone or secure videoconference. Potential participants complete a standardized health questionnaire covering the inclusion and exclusion criteria (cardiovascular, respiratory, neurological, psychiatric, and medication status; smoking; BMI; recent altitude exposure). Older adults additionally complete the TICS‐F to screen global cognitive functioning. Only candidates who meet all inclusion criteria and none of the exclusion criteria are invited to the laboratory.

On the testing day, participants attend a single session lasting approximately 3 h. Upon arrival at the laboratory, they provide written informed consent and complete a brief update of their medical status (e.g., recent illnesses, medications, altitude exposure) to confirm continued eligibility. They are then prepared for physiological recording (ECG, electrodermal activity, pulse oximetry, and automated blood‐pressure monitoring).

Participants are next seated in the normobaric hypoxia chamber with FiO_2_ set to 21%. They first complete a 5‐min seated resting baseline under normoxia. Following this baseline, an acclimatization phase of approximately 40 min begins. During acclimatization, all participants watch the same neutral documentary film with minimal emotional content, allowing them to become accustomed to the chamber environment while limiting strong affective reactions.

For younger adults assigned to the hypoxia condition, FiO_2_ is gradually reduced during the acclimatization phase until arterial oxygen saturation (SpO_2_) stabilizes around 75%–85%, consistent with prior cognitive hypoxia work in healthy adults (e.g., McMorris et al. 2017; Ramírez‐delaCruz et al. 2024) and with ranges typically described as moderate normobaric hypoxia (SpO_2_ = 75%–85%; Timon et al. 2023). SpO_2_, heart rate, blood pressure, and acute mountain sickness symptoms are monitored continuously according to the predefined safety protocol. Younger adults in the normoxia condition and older adults remain at FiO_2_ = 21% throughout acclimatization, with identical monitoring and film viewing. Thus, total time spent in the chamber and exposure to the documentary are matched across groups, and only the oxygen fraction differs between hypoxic and normoxic conditions.

Once the acclimatization phase is complete and SpO_2_ has remained within the target range for at least 5 min, participants complete a second 5‐min seated resting period under their assigned oxygen condition (normoxia vs. hypoxia). This condition‐specific resting period is used to derive resting HRV in the assigned oxygen context. Under the stabilized oxygen condition, participants first complete an experimental task from a related project examining the effects of hypoxia on associative emotional memory; this task uses the same hypoxia protocol and physiological monitoring but is reported elsewhere. Immediately after the memory task and before the emotional image task, and still under the assigned oxygen condition, participants will complete brief state self‐reports of mental fatigue (Mental Fatigue VAS), positive and negative affect using the Positive and Negative Affect Schedule (PANAS; Watson et al. 1988; French version: Gaudreau et al. 2006), and state anxiety using the State form of the State–Trait Anxiety Inventory (STAI‐S; Spielberger 1983; French version: Bruchon‐Schweitzer and Paulhan 1993), to characterize their state at the onset of the coherence task. The same set of state measures will also be administered at the seated normoxic baseline before chamber entry and at the end of the session after return to normoxia, to track changes from baseline. They will then perform the emotional image task followed by the interoceptive accuracy task, in a fixed order as described above. The order is fixed because the chamber protocol requires that the assigned oxygen condition be established and maintained continuously. Counterbalancing task order would make the emotional image task occur at different points in the hypoxic exposure across participants, thereby changing elapsed exposure time, accumulated fatigue, physiological state, and safety‐monitoring demands at task onset. Task order is therefore not procedurally confounded with group assignment because all groups complete the same sequence, but the magnitude of carryover, fatigue, or inter‐task recovery may still differ by oxygen condition. This limitation will be addressed analytically through pre‐task state measures, response‐quality indicators (rating latencies, omission rates), task‐block and trial‐number terms in the trial‐level coherence models, and sensitivity analyses excluding the first emotional‐task block (see Sensitivity analyses). Short self‐paced breaks are provided between blocks and between tasks to limit fatigue and maintain data quality.

At the end of the experimental tasks, the chamber is returned to normoxia (FiO_2_ = 21%). Participants remain seated under observation until SpO_2_ and symptoms have returned to baseline levels and a final safety check (SpO_2_, blood pressure, subjective symptoms) is completed. Physiological sensors are then removed, participants are debriefed, compensated, and free to leave the laboratory.

### 2.12 Statistical Analysis Plan

#### 2.12.1 Software and General Principles

All analyses will be conducted in R (version 4.3.2; R Core Team 2024). Trial‐level GEE models will be fitted with the *geepack* package (Højsgaard et al. 2006). Inference for trial‐level GEE models will use a Mancl–DeRouen small‐sample‐corrected sandwich covariance estimator (Mancl and DeRouen 2001), implemented in R with the *geesmv* package (Wang 2015) or an equivalent validated implementation of modified GEE variance estimators. The conventional robust sandwich estimator from *geepack* will be reported as a robustness check. Regression‐based mediation models, where used, will be implemented with *lavaan* (Rosseel 2012) or the *mediation* package (Tingley et al. 2014). Figures will be created with ggplot2 (Wickham 2016), and data wrangling will rely on the *tidyverse* suite (Wickham et al. 2019). For H3, Bayes factors for group differences in coherence will be computed using standard Bayesian *t*‐test implementations with default Cauchy priors on standardized effect sizes (Rouder et al. 2009) and equivalence tests based on the two one‐sided tests (TOST) procedure will be conducted using the TOSTER package (Lakens 2017). All scripts for data cleaning and analysis will be deposited on OSF at Stage 2 together with an anonymized dataset and codebook to ensure full reproducibility.

#### 2.12.2 Data Quality Checks and Exclusions

Data quality checks will be performed before computing coherence and interoception indices. At the trial level, we will exclude trials with missing or invalid values for the subjective index required by the analysis, or unusable physiological data according to the modality‐specific criteria described above (e.g., excessive ECG artifacts preventing reliable IBI estimation, electrode failures in EDA). For the primary coherence analyses, a valid emotional‐image trial must include a valid arousal rating and usable physiological data for the relevant modality. Missing valence or approach‐motivation ratings will not invalidate a trial for the primary arousal‐based coherence analyses, but the trial will not contribute to exploratory component‐level analyses requiring the missing rating. For the interoception task, trials with missing responses or ECG artifacts preventing reliable R‐peak detection will be excluded from dʹ computation.

For the primary coherence indices, participants will be retained for a given physiological modality if at least 60 valid emotional‐image trials remain after cleaning across valence categories combined. For the primary analyses, a valid trial is defined as a trial with an available arousal rating and usable physiological data for the relevant modality after artifact rejection; for SCR, physiological non‐responses are retained and coded as zero rather than treated as missing. For exploratory component‐level coherence indices, trial validity will be defined with respect to the relevant subjective component: motivational intensity for approach‐based analyses and valence intensity for valence‐based analyses. For valence‐specific coherence indices, a participant will contribute to the analysis for a given stimulus valence only if at least 15 valid trials remain in that valence category out of 40 planned trials. Sample sizes may therefore differ across physiological modalities and valence‐specific analyses, but no participant will be excluded from all coherence analyses solely because one modality or one valence‐specific estimate fails to meet the threshold. For the interoceptive accuracy task, participants with fewer than 60 usable trials out of 80 planned trials will be excluded from analyses involving dʹ. These thresholds are defined a priori to balance data quality with participant retention. We will report retention per physiological modality, separately by group, to allow assessment of whether participant attrition is differential across conditions.

For participant‐level indices (global coherence across all images, valence‐specific coherence, lnRMSSD, interoceptive *d*′, MAIA‐2 total), we will implement a robust outlier rule based on the median absolute deviation (MAD; Leys et al. 2013): values more extreme than ±3 MAD from the group median will be flagged separately within each age/condition group and for each outcome. Outlier handling will be outcome‐specific: participants flagged as outliers for a given index will be excluded from the primary analysis of that index but retained for all other outcomes and for predefined sensitivity analyses including all cases. For valence‐specific coherence, outliers will be identified separately within each valence category so that participants can still contribute valid data for other valences. The ±3 MAD rule and its outcome‐specific application are fixed a priori and will not be tuned based on the observed data.

#### 2.12.3 Operationalization of Emotional Coherence

For each participant, HRdec and SCR values will be standardized within person across valid emotional trials. Subjective affective intensity will be operationalized as the within‐person standardized arousal rating. Z‐scoring within person does not change Pearson correlation coefficients but facilitates comparability across individuals and use in regression‐based models.

We will compute two main coherence indices per participant:

- HR‐based coherence: Pearson correlation between within‐person standardized arousal and within‐person standardized HRdec across all valid emotional trials.
- SCR‐based coherence: Pearson correlation between within‐person standardized arousal and within‐person standardized SCR across all valid emotional trials.

These correlations will be Fisher z‐transformed for group‐level analyses. To explore valence specificity, we will also compute parallel arousal‐based coherence indices within each stimulus valence category (negative, neutral, positive), subject to the minimum‐trials rule described above. Valence‐stratified indices will be treated as secondary outcomes given reduced trial numbers per cell. We will report mean subjective arousal, mean HRdec, and mean SCR magnitude by group to contextualize the coherence indices against the corresponding raw subjective and autonomic reactivity profiles.

Exploratory component‐level coherence indices will additionally be computed using motivational intensity, defined as |approach −50|, and valence intensity, defined as |valence −50|. Each component will be standardized within person across valid emotional trials before correlation with physiological indices. These component‐level analyses will be interpreted as exploratory tests of whether different subjective dimensions show distinct patterns of subjective–physiological coupling.

#### 2.12.4 Primary Person‐Level Analyses ([Link], [Link], [Link])

For each coherence index (HR‐based, SCR‐based), we will fit linear models predicting Fisher z‐transformed coherence from Group (younger–normoxia, younger–hypoxia, older–normoxia). Pre‐registered contrasts will test the three primary hypotheses:

*(aging effect): older–normoxia* vs. *younger–normoxia. We predict lower average coherence in older adults*.

*(hypoxia effect): younger–hypoxia* vs. *younger–normoxia. We predict lower average coherence in younger adults under hypoxia than under normoxia*.

*(partial convergence): younger–hypoxia* vs. *older–normoxia. We predict that coherence in hypoxic younger adults will be numerically similar to that of older adults*.

For H1 and H2, we will conduct two‐sided null‐hypothesis tests with *α* = 0.05 and report unstandardized mean differences, standardized effect sizes (Cohen's d or Hedges' g if sample sizes differ due to exclusion), and 95% confidence intervals (CIs). For H3, we will report the observed effect size with its 95% confidence interval, compute Bayes factors comparing a point‐null model to an alternative allowing non‐zero differences (Rouder et al. 2009), and conduct TOST equivalence tests at the two pre‐specified equivalence bounds (*d* = 0.63 and *d* = 0.35) defined in the Sensitivity analyses section. Partial convergence will be considered supported only if TOST is significant at the more conservative *d* = 0.35 bound; a non‐significant TOST will be reported as failure to establish equivalence rather than evidence for a meaningful difference.

#### 2.12.5 Trial‐Level GEE Models

To complement person‐level coherence indices, we will model trial‐level data using GEEs with participant as the clustering factor, an exchangeable working correlation, and the Mancl–DeRouen small‐sample‐corrected covariance estimator described above. Separate models will be fitted for within‐person standardized HRdec and SCR as dependent variables.

To distinguish trial‐level coupling from differences in overall arousal level, subjective arousal will be decomposed from the raw 0–100 arousal ratings into within‐person and between‐person components (Curran and Bauer 2011). The within‐person arousal component will be computed as trial‐level arousal minus the participant's mean arousal across valid emotional trials. The between‐person arousal component will be the participant's mean arousal across valid emotional trials, grand‐mean centered. If predictors are standardized for model comparability, standardization will be applied after this within‐person/between‐person decomposition so that the between‐person arousal term is preserved.

Main predictors will include Group, within‐person arousal, between‐person arousal, stimulus Valence, block, trial number centered within block, and the Group × within‐person arousal interaction. The focal GEE tests will be the Group × within‐person arousal interactions. These terms estimate whether trial‐to‐trial deviations in a participant's own arousal are more or less strongly coupled with autonomic reactivity across groups, while the between‐person arousal term separates coupling changes from group differences in overall arousal level. Block and trial‐number terms are included to capture habituation, time‐on‐task, or progressive hypoxia‐related changes during the emotional task. Stimulus Valence will be dummy‐coded to allow planned contrasts, and Group × within‐person arousal × Valence interactions will be treated as exploratory. These GEE models will be treated as secondary, convergent confirmatory tests of [Link], [Link], [Link] at the trial level, complementing the person‐level coherence indices.

#### 2.12.6 SCR‐Based Coherence: Two‐Part Trial‐Level Specification

Because SCR magnitude jointly reflects response occurrence and response size, SCR‐based coherence will be additionally analyzed using a two‐part model that separates these components.

The first component (i.e., the response‐occurrence model) will estimate whether subjective arousal is coupled to the probability of producing a detectable SCR on a trial. We will fit a logistic GEE with a binary outcome (SCR detected: yes/no), Subject as the clustering unit, an exchangeable working correlation structure, and predictors including within‐person arousal, between‐person arousal, Group, the Group × within‐person arousal interaction, local pre‐stimulus SCL, stimulus Valence, block, and trial number centered within block. Local pre‐stimulus SCL will be included to evaluate whether tonic electrodermal activation at stimulus onset is associated with the likelihood of detecting a phasic SCR. When feasible, local pre‐stimulus SCL will be decomposed into a within‐person component, indexing trial‐to‐trial tonic fluctuations, and a participant‐mean component, indexing each participant's average tonic level during the task. The Group × within‐person arousal interaction tests whether the within‐person association between subjective arousal and SCR occurrence differs across groups.

The second component (i.e., the conditional‐amplitude model) will estimate, among trials on which a detectable SCR occurred, whether subjective arousal is coupled to SCR amplitude. We will fit a linear GEE on natural‐log‐transformed SCR amplitude with the same predictor structure. If the number of detected SCRs is insufficient in one or more groups to support stable conditional‐amplitude modeling, this analysis will be reported descriptively and interpreted cautiously.

For participant‐level analyses, tonic‐adjusted SCR coherence will be computed as the partial correlation between trial‐level subjective arousal and trial‐level SCR magnitude, controlling for trial‐level local pre‐stimulus SCL within each participant. Equivalently, SCR will be regressed on local pre‐stimulus SCL within each participant and the residuals correlated with subjective arousal. This adjustment will be interpreted as partialling out the linear association between SCR magnitude and local tonic baseline; it will not be interpreted as fully correcting tonic–phasic masking or recovering undetectable phasic responses.

#### 2.12.7 Mechanism‐Oriented Analyses: Resting HRV and Interoceptive Accuracy

In secondary, mechanism‐oriented analyses inspired by PHEA, we will examine whether group differences in emotional coherence are statistically related to differences in autonomic capacity and interoceptive accuracy under the assigned oxygen condition. For each primary coherence index, we will fit regression models including Group and either lnRMSSD (condition‐specific) or interoceptive dʹ as predictors. We will first test whether Group predicts lnRMSSD and dʹ (path a) and whether lnRMSSD and dʹ predict coherence over and above Group (path b).

If both conditions are met, we will estimate indirect effects of Group on coherence via lnRMSSD and via dʹ using regression‐based mediation models with non‐parametric bootstrapped confidence intervals (10,000 resamples). An indirect effect will be considered statistically supported if the 95% bias‐corrected bootstrap CI excludes zero. Because these data are cross‐sectional and temporal ordering is not experimentally manipulated, we will interpret such indirect paths as statistical evidence consistent with, but not definitive proof of, the hypothesized mechanisms. These mediation analyses will not be treated as tests of additional primary hypotheses; rather, they will be used to assess whether the pattern of group differences in coherence aligns with the idea that reduced autonomic capacity and degraded interoceptive precision under hypoxia and aging contribute to weaker subjective–physiological coupling.

#### 2.12.8 Exploratory Role of Trait Interoceptive Sensibility

Trait interoceptive sensibility (MAIA‐2 total score) will be examined exploratorily. Specifically, we may include MAIA‐2 as a covariate in the mediation models above to test whether any association between lnRMSSD or interoceptive dʹ and coherence persists after accounting for stable interoceptive style. We may also explore whether MAIA‐2 moderates the association between dʹ and coherence (i.e., whether individuals with higher trait sensibility show stronger links between state interoceptive accuracy and emotional coherence). Such moderation analyses will be clearly labeled as exploratory and interpreted primarily through effect sizes and confidence intervals, without adjustment for multiple comparisons.

#### 2.12.9 Sensitivity Analyses

To assess the robustness of our primary findings, we will conduct several sensitivity analyses. First, we will re‐run the main models after excluding participants flagged as outliers on the relevant coherence index to verify that conclusions do not depend on extreme values. Second, we will repeat the analyses using rank‐based coherence indices (Spearman correlations) instead of Pearson correlations to evaluate sensitivity to distributional assumptions. Third, we will examine whether results hold when analyzing HR‐based and SCR‐based coherence separately for each valence category (negative, neutral, positive); these valence‐stratified analyses will be clearly labeled as exploratory given reduced statistical power. Fourth, we will test whether including baseline normoxic lnRMSSD as a covariate alters the pattern of group differences in coherence, which will inform whether condition‐specific autonomic changes are more relevant than stable trait‐like autonomic capacity for explaining any observed group differences in coherence. Fifth, to evaluate whether group differences in coherence are robust to plausible non‐specific influences on subjective–physiological coupling, three sets of variables will be summarized by group: state measures collected immediately before the emotional image task (Mental Fatigue VAS, PANAS positive and negative affect, STAI‐S), task‐embedded response‐quality and attentional engagement indicators (median rating latency, omission rate), and baseline individual‐difference measures (BMI, ERQ Reappraisal and Suppression subscales, MAIA‐2 total score, and MAIA‐2 Emotional Awareness subscale). If any of these variables shows a between‐group difference at *p* < 0.10 or |d| ≥ 0.30, the primary person‐level coherence model will be re‐estimated with that variable added as a mean‐centered covariate, one covariate at a time, to limit overfitting given the planned sample size, while keeping the primary model specification otherwise unchanged. Sixth, to evaluate whether potential carryover from the preceding memory task or early‐task instability affects the primary coherence findings, we will repeat the primary coherence analyses after excluding the first block of the emotional image task. This analysis will be interpreted as a sensitivity check. If the results are materially attenuated or change direction after exclusion of the first block, the primary findings will be interpreted cautiously as potentially influenced by carryover or early adaptation to the emotional‐task context. Finally, to address the possibility that elevated tonic electrodermal activation under hypoxia reduces the detectability or dynamic range of phasic SCR responding, SCR‐based analyses will be accompanied by summaries of tonic SCL, local pre‐stimulus SCL, SCR magnitude including zero‐coded non‐responses, SCR response frequency, SCR amplitude conditional on response detection, and within‐person SCR variability by group and block. SCR‐based coherence will be re‐estimated (i) using the two‐part response‐occurrence and conditional‐amplitude models described in the Statistical analysis plan, with local pre‐stimulus SCL included as a covariate; (ii) using participant‐level partial correlations between subjective arousal and SCR magnitude controlling for local pre‐stimulus SCL; and (iii) using alternative fixed SCR detection thresholds of 0.01, 0.02, and 0.05 μS to evaluate whether conclusions depend on the SCR detection criterion. Two patterns would point toward tonic–phasic confounding. First, if hypoxia is associated with elevated tonic SCL but reduced SCR response frequency or magnitude. Second, if SCR‐based coherence effects attenuate after adjustment for local pre‐stimulus SCL, or differ between the response‐occurrence and conditional‐amplitude models. Under either pattern, SCR‐based coherence effects will be interpreted cautiously as potentially reflecting tonic–phasic compression or reduced phasic detectability, rather than reduced electrodermal sympathetic engagement per se. If SCR‐based coherence effects remain similar after adjustment for local pre‐stimulus SCL and are also observed in the conditional‐amplitude model, the findings will support interpretation in terms of altered stimulus‐locked phasic reactivity. If results differ across SCR detection thresholds, SCR‐based conclusions will be interpreted as threshold‐sensitive.

## Author Contributions

**Grégoire Millet:** writing – review and editing, resources, methodology. **Ulrike Rimmele:** resources, writing – review and editing, project administration, methodology. **Pierrick Laulan:** conceptualization, methodology, resources, writing – original draft, writing – review and editing, project administration, funding acquisition.

## Funding

This work was supported by the Spark grant from the Swiss National Science Foundation (SNSF; grant number 237841/1). The funder has no role in the study design, data collection, analysis, interpretation, or decision to submit the manuscript for publication.

### Disclosures

*Preregistration*: This Stage 1 Registered Report has received in‐principle acceptance at Psychophysiology. The introduction, methods, and analysis plan described here constitute the preregistered protocol. Following in‐principle acceptance, the approved protocol will be archived on OSF, and any deviations from the registered plan will be transparently documented in the Stage 2 report. *Data*: Upon acceptance of the Stage 2 manuscript, de‐identified data sufficient to reproduce all reported analyses will be made publicly available on OSF. Data will be shared in a tidy, analysis‐ready format, accompanied by a detailed codebook. *Materials*: Because of copyright restrictions, the original IAPS images cannot be redistributed. However, we will share the full list of picture identifiers, all trial‐wise stimulus properties (e.g., normative valence and arousal ratings, low‐level visual features), and all experimental scripts (e.g., task code for the emotional image and interoception tasks) on OSF. *Code*: All analysis scripts (R code for data cleaning, computation of coherence indices, and statistical models) will be uploaded to OSF at Stage 2, allowing full reproducibility of the reported results.

## Ethics Statement

The study protocol has been submitted to the Cantonal Research Ethics Committee, Vaud (CER‐VD) and will be conducted in accordance with the Declaration of Helsinki. Any modifications requested by the committee will be implemented via formal amendments before data collection begins. At the time of this Stage 1 submission, no data have been collected.

## Conflicts of Interest

The authors declare no conflicts of interest.

## Data Availability

Data sharing not applicable to this article as no datasets were generated or analysed during the current study.
